# Carbon Monoxide Releasing Molecule-2-Upregulated ROS-Dependent Heme Oxygenase-1 Axis Suppresses Lipopolysaccharide-Induced Airway Inflammation

**DOI:** 10.3390/ijms20133157

**Published:** 2019-06-28

**Authors:** Chih-Chung Lin, Li-Der Hsiao, Rou-Ling Cho, Chuen-Mao Yang

**Affiliations:** 1 Department of Anesthetics, Chang Gung Memorial Hospital at Linkuo, and College of Medicine, Chang Gung University, Kwei-San, Tao-Yuan 33302, Taiwan; 2 Department of Physiology and Pharmacology and Health Aging Research Center, College of Medicine, Chang Gung University, 259 Wen-Hwa 1 Road, Kwei-San, Tao-Yuan 33302, Taiwan; 3 Research Center for Chinese Herbal Medicine and Research Center for Food and Cosmetic Safety, College of Human Ecology, Chang Gung University of Science and Technology, Tao-Yuan 33302, Taiwan

**Keywords:** CORM-2, NADPH oxidase, ROS, AP-1, HO-1

## Abstract

The up-regulation of heme oxygenase-1 (HO-1) is mediated through nicotinamaide adenine dinucleotide phosphate (NADPH) oxidases (Nox) and reactive oxygen species (ROS) generation, which could provide cytoprotection against inflammation. However, the molecular mechanisms of carbon monoxide-releasing molecule (CORM)-2-induced HO-1 expression in human tracheal smooth muscle cells (HTSMCs) remain unknown. Here, we found that pretreatment with CORM-2 attenuated the lipopolysaccharide (LPS)-induced intercellular adhesion molecule (ICAM-1) expression and leukocyte count through the up-regulation of HO-1 in mice, which was revealed by immunohistochemistrical staining, Western blot, real-time PCR, and cell count. The inhibitory effects of HO-1 by CORM-2 were reversed by transfection with HO-1 siRNA. Next, Western blot, real-time PCR, and promoter activity assay were performed to examine the HO-1 induction in HTSMCs. We found that CORM-2 induced HO-1 expression via the activation of protein kinase C (PKC)α and proline-rich tyrosine kinase (Pyk2), which was mediated through Nox-derived ROS generation using pharmacological inhibitors or small interfering ribonucleic acids (siRNAs). CORM-2-induced HO-1 expression was mediated through Nox-(1, 2, 4) or p47*^phox^*, which was confirmed by transfection with their own siRNAs. The Nox-derived ROS signals promoted the activities of extracellular signal-regulated kinase 1/2 (ERK1/2). Subsequently, c-Fos and c-Jun—activator protein-1 (AP-1) subunits—were up-regulated by activated ERK1/2, which turned on transcription of the HO-1 gene by regulating the HO-1 promoter. These results suggested that in HTSMCs, CORM-2 activates PKCα/Pyk2-dependent Nox/ROS/ERK1/2/AP-1, leading to HO-1 up-regulation, which suppresses the lipopolysaccharide (LPS)-induced airway inflammation.

## 1. Introduction

Heme oxygenase (HO), a rate-limiting enzyme, metabolizes heme to biliverdin-IXα, ferrous iron, and carbon monoxide (CO). These secondary products are involved in the regulation of different physiological processes. HO-1, a member of the HO family [1,2], is inducible and directly protects various organs from oxidative damages [1,3]. Biliverdin-IXα is converted to the endogenous radical scavenger bilirubin-IXα and has anti-inflammatory properties [4,5]. The ferrous iron is rapidly sequestered with ferritin to function additional antioxidant and anti-apoptotic effects [5,6]. Moreover, CO has been shown to exert anti-apoptotic and anti-inflammatory effects that are mediated via HO-1 up-regulation [4,7,8]. However, several pro-inflammatory cytokines and oxidative stresses can also trigger HO-1 expression [9,10,11]. For example, HO-1 is also induced by various factors in the airway cells of asthmatic patients [12]. Accumulating evidence concerning HO-1/CO-dependent cytoprotection elicits the mechanisms involved in the modulation of the inflammatory responses, including the down-regulation of pro-inflammatory mediators, atherosclerosis, ischemia-reperfusion systems, and airway disorders [8,13,14] 

Nicotinamaide adenine dinucleotide phosphate (NADPH) oxidase (Nox)-derived reactive oxygen species (ROS) generation has been approved to regulate either the expression of inflammatory or anti-inflammatory mediators in the airway and pulmonary diseases [15]. Excessive ROS production can regulate the expression of various inflammatory genes during airway disorders [15,16]. In contrast, low levels of ROS contribute to maintain cellular redox homeostasis under physiological conditions [15]. Several studies indicate that the exogenous application of CO and HO-1 can protect against oxidative stress and hyperoxic injury in the various organs [17,18]. It has also been reported that the up-regulation of HO-1 via the Nox/ROS formation is induced by lipopolysaccharide (LPS) or cytokines [8,19]. In addition, our previous reports have indicated that the Nox/ROS system is a key player for HO-1 expression induced by lipotechoic acid (LTA) and cigarette smoke particle extract (CSPE) in human tracheal smooth muscle cells (HTSMCs) [5,20]. Our previous studies and others have demonstrated that the carbon monoxide-releasing molecule (CORM)-2 mediates the Nox-dependent ROS generation in astrocytes [21,22] and human bronchial smooth muscle cells [23]. CORMs have been confirmed to provide the exogenous CO source and induce HO-1 expression in several cell types [8,9,24]. However, the roles of Nox/ROS involved in CORM-2-induced HO-1 expression were still unknown. HO-1 expression is regulated by various intracellular signaling pathways, such as ROS, growth factor receptors, non-receptor tyrosine kinases such as Pyk2, or mitogen-activated protein kinases (MAPKs) [25,26]. In our previous study, CORM-2 has been shown to induce HO-1 expression via c-Src/epidermal growth factor receptor (EGFR)/phosphoinositide 3-kinase (PI3K)/Akt/c-Jun N-terminal kinase 1/2 (JNK1/2) and p38 MAPK pathways in HTSMCs [27]. We also noticed that many stress-activated response elements on the upstream region of the HO-1 promoter, such as nuclear factor erythroid 2-related factor 2 (Nrf2) and activator protein 1 (AP-1), are involved in the expression of HO-1 in response to oxidative stresses [28,29]. Therefore, whether an alternative Nox/ROS-mediated pathway involved in HO-1 expression induced by CORM-2 has yet to be investigated in HTSMCs.

Besides, several reports have shown that administration with low concentrations of CO or pharmacological application of CORMs can also confer protective effects in the models of inflammatory responses and tissue injury [26,30,31]. Accumulating evidence has indicated that CORM-2-liberated CO reduces inflammatory responses in sepsis by interfering with nuclear factor (NF)-κB activation [32]. Moreover, the overexpression of HO-1 by cobalt protoporphyrin (CoPPIX) can reduce tumor necrosis factor (TNF) α-induced oxidative stress and airway inflammation [7]. Therefore, CORM-2-induced HO-1 gene expression could prevent inflammatory responses. However, the detailed mechanisms by which CORM-2 induced HO-1 expression in HTSMCs are still unclear. Our results demonstrated that CORM-2-induced HO-1 expression is mediated via protein kinase C (PKC) α/proline-rich tyrosine kinase 2 (Pyk2)-dependent Nox/ROS generation linking to the ERK1/2-mediated activation of AP-1 in HTSMCs, which could protect against the lipopolysaccharide (LPS)-induced airway inflammatory diseases. 

## 2. Results

### 2.1. CORM-2 Inhibits LPS-Induced Lung Inflammation in Mice 

**Figure 1:**
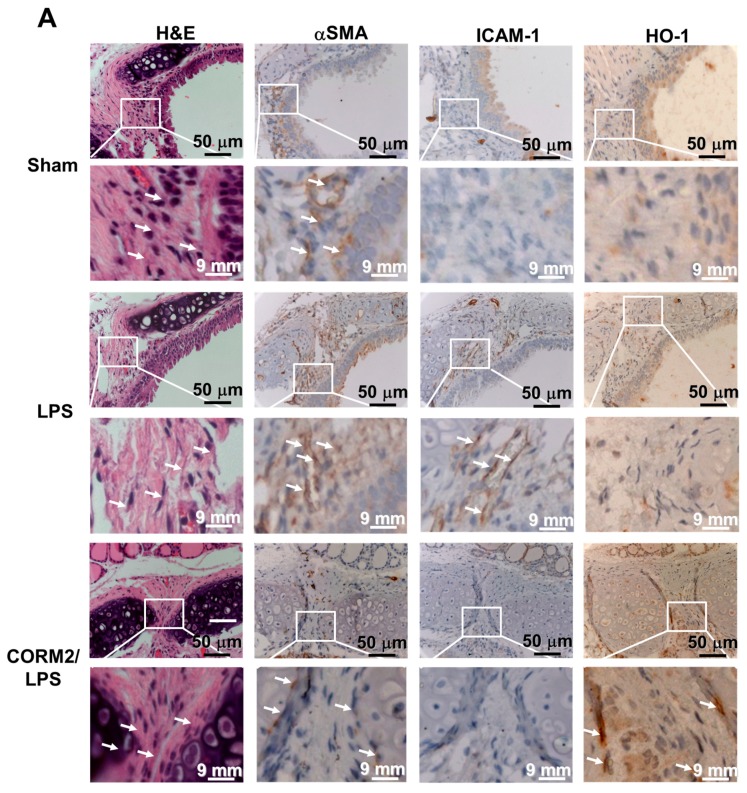

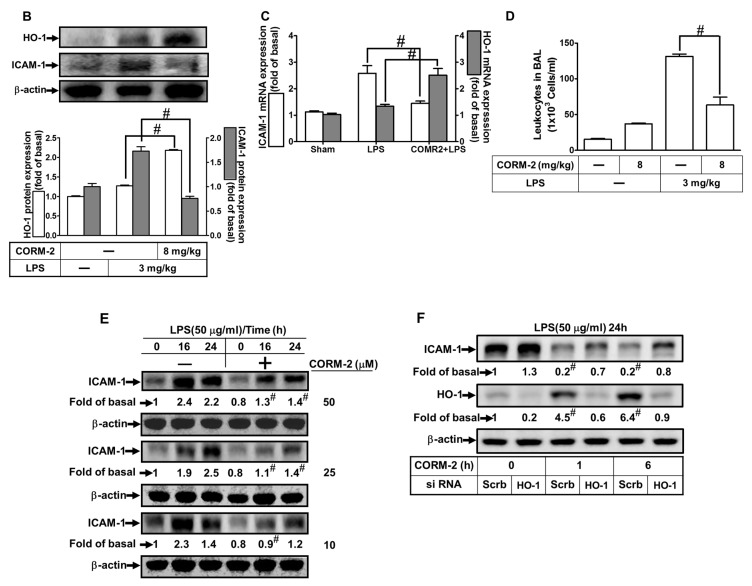
Induction of heme oxygenase (HO-1) by carbon monoxide-releasing molecule (CORM-2) suppresses intercellular adhesion molecule-1 (ICAM-1) expression and leukocyte infiltration in HTSMCs and mice. Institute of Cancer Research (ICR) mice were pretreated with CORM-2 (8 mg/kg of body weight) for 24 h, and then treated with lipopolysaccharide (LPS) (3 mg/kg of body weight). (**A**) H (hematoxylin) & E (Eosin) and immunohistochemical staining for α-smooth muscle actin (α-SMA), ICAM-1, and HO-1 in serial section of the airway tissues from sham [0.1 mL of DMSO–PBS (phosphate-buffered saline) (1:100) with 0.1% (*w*/*v*) BSA (bovine serum albumin) treated mice], LPS (LPS-injected mice) and CORM-2 + LPS mice. The arrows indicate tracheal smooth muscle cells displayed with ICAM-1 and HO-1 expression. (**B**,**C**) Airway tissues were homogenized to extract proteins and mRNAs (messenger ribonucleic acids), and analyzed by (**B**) Western blot and (**C**) real-time PCR to determine the levels of HO-1, ICAM-1, and β-actin (served as an internal control) protein and mRNA expression, respectively. (**D**) BAL fluid was collected to count the number of leukocytes infiltration. (**E**) Human tracheal smooth muscle cells (HTSMCs) were pretreated without or with various concentrations of CORM-2 for 1 h and then incubated with LPS (50 μg/mL) for the indicated time periods. The levels of ICAM-1 and β-actin were determined by Western blot. (**F**) Cells were transfected with scrambled (Scrb) or HO-1 siRNA (small interfering ribonucleic acid), incubated with CORM-2 (50 μM) for 1 or 6 h, and then stimulated with LPS (50 μg/mL) for 16 h. The levels of ICAM-1, HO-1, and β-actin protein were determined by western blot. Data are expressed as mean ± SEM of five independent experiments (*n* = 5). ^#^ *p* < 0.05, as compared with the mice exposed to the indicated reagents. Data analysis and processing are described in the section “Statistical Analysis of Data”.

CORM-2 has been shown to protect against inflammatory responses induced by various insults [4,7,8]. First, we investigated the anti-inflammatory effects of CORM-2 on LPS-induced lung inflammation. We observed that LPS markedly induced intercellular adhesion molecule-1 (ICAM-1) expression, which was attenuated by CORM-2 via HO-1 expression, as determined by immunohistochemistry staining (Figure 1A). Bronchoalveolar lavage (BAL) fluid was collected to determine the number of leukocytes, and airway tissues were harvested to study the levels of protein and mRNA expression. As shown in Figure 1B,D, LPS significantly enhanced ICAM-1 protein expression and leukocytes count in BAL fluid. LPS also significantly induced ICAM-1 messenger ribonucleic acid (mRNA) expression (Figure 1C). In addition, pretreatment with CORM-2 also inhibited the LPS-induced ICAM-1 protein expression in HTSMCs (Figure 1E). Further, we confirmed the inhibitory effects of HO-1 induction by CORM-2 on the LPS-up-regulated ICAM-1 expression by transfection with HO-1 siRNA. We found that CORM-2 attenuated the LPS-induced ICAM-1 expression, which was partially reversed by transfection with HO-1 siRNA (Figure 1F). All of these LPS-mediated responses were attenuated by CORM-2 via the up-regulation of HO-1 in the airway tissues of mice (Figure 1). These results suggested that the up-regulation of HO-1 by CORM-2 protects airway tissues against the LPS-mediated inflammatory responses.

### 2.2. ROS Participate in CORM-2-Induced HO-1 Expression

**Figure 2:**
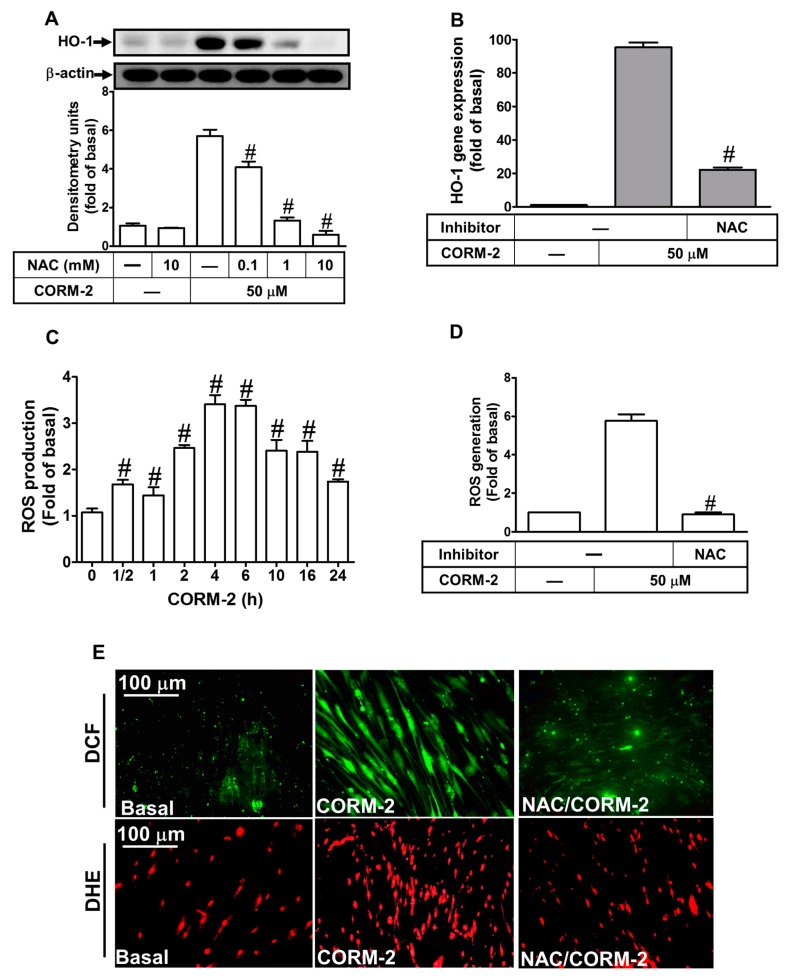

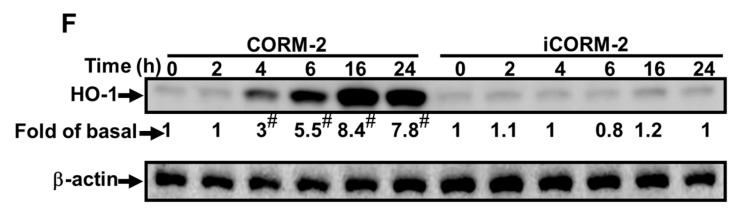
Reactive oxygen species (ROS) are required for CORM-2-induced HO-1 expression in HTSMCs. (**A**) Cells were pretreated with N-acetyl cysteine (NAC) for 1 h and then incubated with CORM-2 for 24 h. The protein levels of HO-1 and β-actin (served as an internal control) were determined by Western blot. (**B**) Cells were pretreated with NAC (10 mM) for 1 h, and then incubated with CORM-2 for 6 h. The mRNA expression of HO-1 was determined by real-time PCR. (**C**) Cells were incubated with the 2',7'-dichlorofluorescin diacetate (DCF-DA) (5 μM) for 45 min, followed by stimulation with 50 μM of CORM-2 for the indicated time intervals. (**D**) Cells were pretreated without or with NAC (10 mM) for 1 h before exposure to CORM-2 for 6 h. (**C**,**D**) The fluorescence intensity of cells was determined. (**E**) DCF-DA, and dihydroethidium (DHE) staining, cells were treated with CORM-2 for 6 h in the absence or presence of NAC (10 mM). The fluorescence images were observed by a fluorescence microscope. Image of fluorescence microscope, 400×. (**F**) Cells were treated with 50 μM of CORM-2 or iCORM-2 for the indicated time intervals. The protein levels of HO-1 were determined by Western blot. Data are expressed as mean ± SEM of five independent experiments (*n* = 5). ^#^ *p* < 0.05, as compared with the cells exposed to vehicle (**C**,**F**) or CORM-2 (**A**,**B**,**D**) alone. Data analysis and processing are described in the section “Statistical Analysis of Data”.

Low levels of ROS have been shown to contribute to maintain cellular redox homeostasis and protect cells against oxidative stress through the up-regulation of HO-1 [8,19]. To examine whether ROS participate in HO-1 induction in HTSMCs, a ROS scavenger N-acetyl cysteine (NAC) was used for this purpose. We found that pretreatment with NAC concentration-dependently attenuated the CORM-2-induced both of HO-1 protein (Figure 2A) and mRNA expression (Figure 2B), suggesting the involvement of ROS in the CORM-2-induced HO-1 expression. Next, we evaluated whether CORM-2 stimulated ROS generation and the scavenging efficacy of NAC. Our results indicated that CORM-2 stimulated ROS generation in a time-dependent manner with a maximal response within 4 h (Figure 2C), which was markedly attenuated by pretreatment with 10 mM of NAC (Figure 2D), indicating that NAC can efficiently scavenge ROS in HTSMCs. These results were further supported by the data of 2′,7′-chloromethyl 2’,7’-dichloro fluorescein diacetate (CMH2DCF-DA; for H_2_O_2_) and dihydroethidium (DHE; for O_2_^−^) fluorescence images observed under a fluorescent microscope (Figure 2E). Pretreatment of HTSMCs with NAC (10 mM) significantly reduced CORM-2-stimulated H_2_O_2_ and O_2_^−^ generation. In addition, treatment with an inactive form of CORM-2 [iCORM-2, ruthenium (III) chloride, RuCl_3_] [33] failed to induce HO-1 expression (Figure 2F). These results concluded that ROS generation stimulated by CORM-2 contributes to up-regulation of HO-1 in HTSMCs.

### 2.3. Nox-Derived ROS Generation Contributes to CORM-2-Induced HO-1 Expression

**Figure 3:**
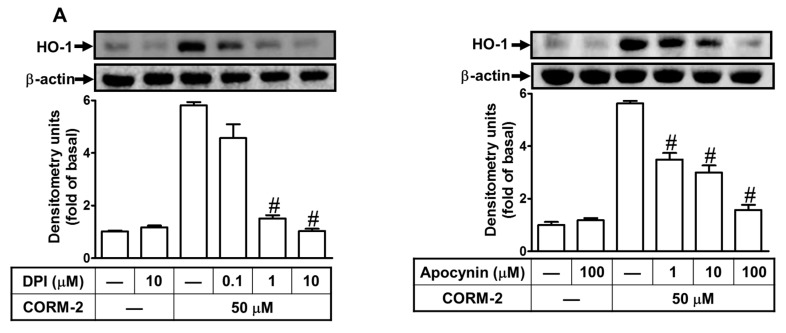

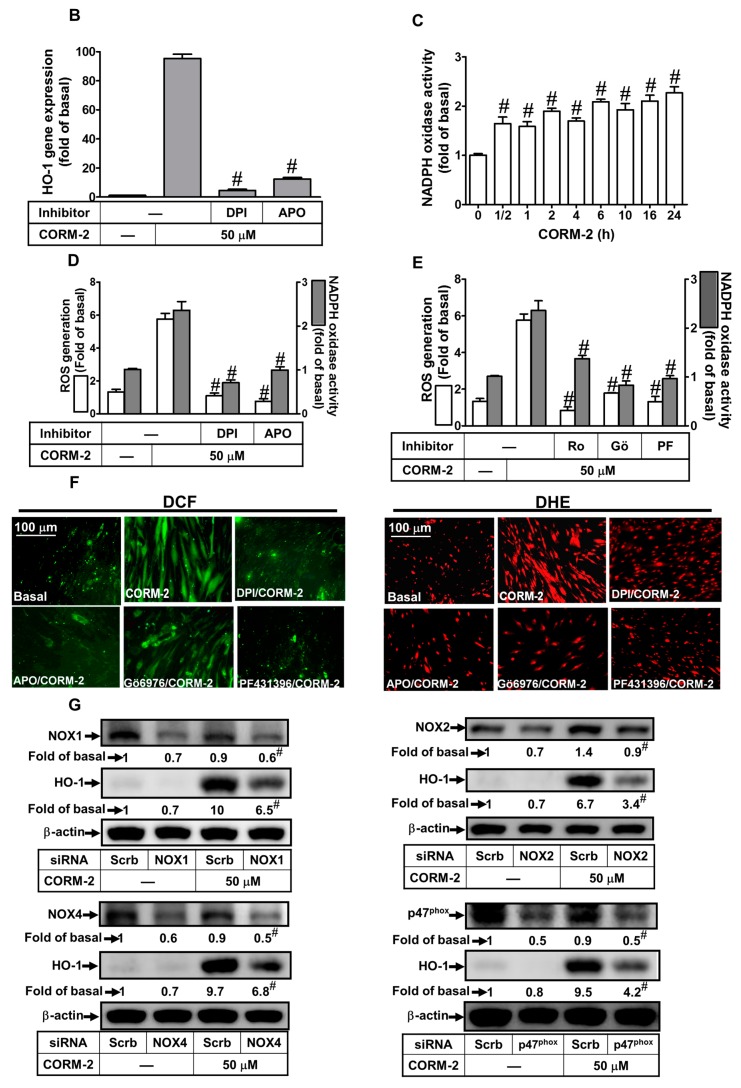
Nicotinamaide adenine dinucleotide phosphate (NADPH) oxidase-dependent ROS generation contributes to CORM-2-induced HO-1 expression in HTSMCs. (**A**) Cells were pretreated without or with diphenyleneiodonium (DPI) or apocynin (APO) for 1 h before exposure to CORM-2 for 24 h. The protein levels of HO-1 and β-actin (served as an internal control) were determined by Western blot. (**B**) Cells were pretreated with DPI (10 μM) or APO (100 μM) for 1 h, and then incubated with CORM-2 for 6 h. The mRNA expression of HO-1 was determined by real-time PCR. (**C**) Cells were incubated with CORM-2 (50 μM) for the indicated time intervals. The Nox activity was analyzed. (**D**,**E**) Cells were pretreated without or with (**D**) DPI (10 μM) or APO (100 μM), and (**E**) Ro31-8220 (10 μM), Gö6976 (10 μM), or PF431396 (10 μM) for 1 h, and then incubated with CORM-2 for 6 h. The Nox activity and ROS generation were analyzed. (**F**) CMH2, DCF-DA, and DHE staining, cells were treated with CORM-2 for 6 h in the absence or presence of DPI (10 μM), APO (100 μM), Gö6976 (10 μM), or PF431396 (10 μM). The fluorescence images were detected by a fluorescence microscope. Image of fluorescence microscope, 400×. (**G**) Cells were transfected with either scrambled (Scrb), Nox-(1,2,4), or p47^phox^ siRNA, and then incubated with CORM-2 for 24 h. The levels of Nox-(1,2,4), p47*^phox^*, HO-1, and β-actin (served as an internal control) protein were determined by Western blot. Data are expressed as mean ± SEM of five independent experiments (*n* = 5). ^#^ *p* < 0.05, as compared with the cells exposed to vehicle (**C**,**G**) or CORM-2 alone (**A**,**B**,**D**,**E**). Data analysis and processing are described in the section “Statistical Analysis of Data”.

CORM-2 mediates the Nox-dependent ROS generation, leading to HO-1 expression [8,9,24]. Thus, the roles of Noxs in the ROS-dependent HO-1 expression were investigated. The inhibitors of Nox (diphenyleneiodonium, DPI) and p47*^phox^* (apocynin, APO) were used to investigate whether Noxs mediated ROS-dependent HO-1 expression in CORM-2-treated HTSMCs. As shown in Figure 3A,B, pretreatment with either DPI or APO significantly attenuated CORM-2-induced both HO-1 protein and mRNA expression. To further investigate whether CORM-2 stimulates Nox activity, as shown in Figure 3C, CORM-2 time-dependently stimulated Nox activity, significantly increased within 30 min and sustained up to 24 h, which was blocked by pretreatment with either DPI (10 μM) or APO (100 μM) (Figure 3D, grey bars), accompanied with inhibiting the ROS generation induced by CORM-2 (Figure 3D, open bars). These results were further supported by the data of DCF (dichlorofluorescein) (for H_2_O_2_) and DHE (for O_2_^−^) fluorescence images observed under a fluorescent microscope (Figure 3F), suggesting that CORM-2-stimulated Nox-derived ROS generation contributes to HO-1 expression. Moreover, we found that Nox/ROS generation stimulated by CORM-2 was mediated via Nox(1,2,4) or p47*^phox^*, which was confirmed by using their own siRNAs in HTSMCs (Figure 3G). 

### 2.4. CORM-2 Induces HO-1 Expression via PKCα 

**Figure 4:**
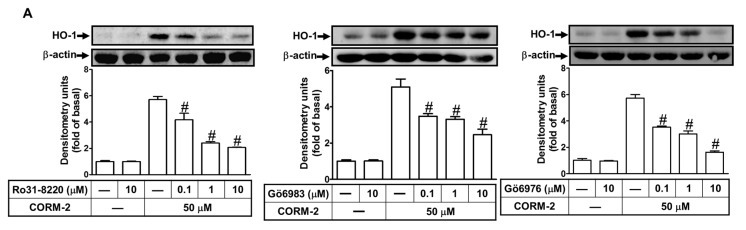

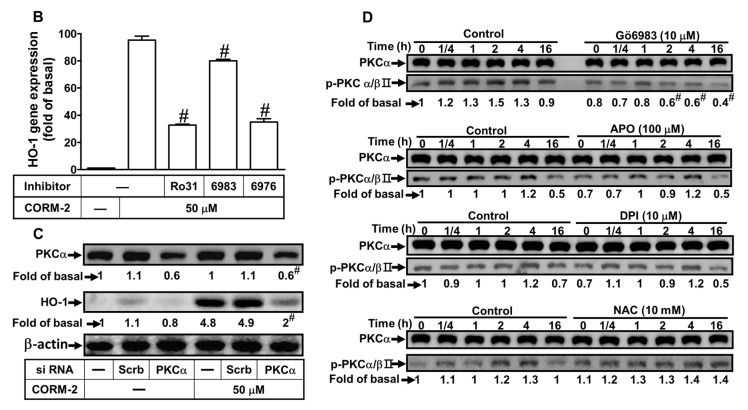
CORM-2 induces HO-1 expression via a PKCα-dependent pathway in HTSMCs. (**A**) Cells were pretreated with Ro31-8220, Gö6983, or Gö6976 for 1 h, and then incubated with CORM-2 (50 μM) for 24 h. The protein levels of HO-1 and β-actin (served as an internal control) were determined by Western blot. (**B**) Cells were pretreated with Ro31-8220 (10 μM), Gö6983 (10 μM), or Gö6976 (10 μM) for 1 h, and then incubated with CORM-2 for 6 h. The mRNA expression of HO-1 was determined by real-time PCR. (**C**) Cells were transfected with either scrambled (Scrb) or protein kinase C (PKCα) siRNA, and then incubated with CORM-2 for 24 h. The levels of PKCα, HO-1, and β-actin (served as an internal control) protein were determined by Western blot. (**D**) Cells were pretreated without or with Gö6983 (10 μM), APO (100 μM), DPI (10 μM), or NAC (10 mM) for 1 h, and then incubated with CORM-2 for the indicated time intervals. The levels of phospho-PKCα and PKCα were determined by Western blot. Data are expressed as mean ± SEM of five independent experiments (*n* = 5). ^#^ *p* < 0.05, as compared with the cells exposed to CORM-2 alone. Data analysis and processing are described in the section “Statistical Analysis of Data”.

Our previous report and the others also indicate that PKCs are involved in HO-1 expression in brain astrocytes [25]. Thus, we investigated whether PKC members are involved in the CORM-2-induced HO-1 expression. We found that pretreatment of HTSMCs with Ro31-8220 (a pan-PKC inhibitor) concentration-dependently attenuated the HO-1 induction by CORM-2 (Figure 4A). Next, to determine which PKC isoforms, PKCα especially, mediate CORM-2-induced HO-1 expression, two selective PKCα inhibitors (Gö6976 and Gö6983) were used for these purposes. The usage of Gő6983, an ATP-competitive bisindolylmaleimide PKC inhibitor, blocks the PKC phosphorylation [34]. Our previous report also indicates that Gő6983 inhibits PKCα/βII phosphorylation stimulated by thrombin in SK–N–SH (human neuroblastoma) cells [35]. We found that pretreatment with either Gö6976 or Gö6983 concentration-dependently blocked CORM-2-induced HO-1 expression in HTSMCs (Figure 4A), indicating that PKCα was involved in CORM-2-induced HO-1 expression. Moreover, pretreatment with Ro31-8220 (10 μM), Gö6976 (10 μM), or Gö6983 (10 μM) significantly inhibited CORM-2-induced HO-1 mRNA expression (Figure 4B). The role of PKCα in CORM-2-induced HO-1 expression was further confirmed by transfection with PKCα siRNA, which significantly knocked down PKCα protein and blocked the CORM-2-induced HO-1 expression (Figure 4C). In addition, the activation of PKCα/βII in CORM-2-induced responses was confirmed by determining their phosphorylation. As shown in Figure 4D, CORM-2 time-dependently stimulated PKCα/βII phosphorylation with a maximal response within 2–4 h, which was attenuated by pretreatment with Gö6983 (10 μM), but not by pretreatment with APO, DPI, or NAC, indicating that PKCα/βII are the upstream components of Nox/ROS in HO-1 expression. This note was also supported by the results that pretreatment with either Ro31-8220 or Gö6976 inhibited the CORM-2-stimulated Nox activity and ROS generation (Figure 3E,F). These results suggested that HO-1 expression induced by CORM-2 is mediated via PKCα/βII-dependent Nox activation and ROS generation in HTSMCs.

### 2.5. Involvement of Pyk2 in CORM-2-Induced HO-1 Expression

**Figure 5:**
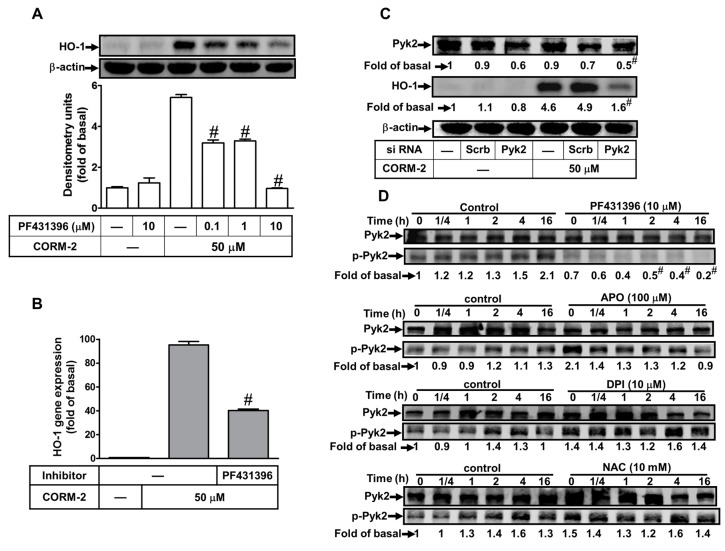
Proline-rich tyrosine kinase 2 (Pyk2) is involved in CORM-2-induced HO-1 expression in HTSMCs. (**A**) Cells were pretreated with PF431396 for 1 h, and then incubated with CORM-2 for 24 h. The protein levels of HO-1 and β-actin (served as an internal control) were determined by western blot. (**B**) Cells were pretreated with PF431396 (10 μM) for 1 h, and then incubated with CORM-2 for 6 h. The mRNA expression of HO-1 was determined by real-time PCR. (**C**) Cells were transfected with either scrambled (Scrb) or Pyk2 siRNA, and then incubated with CORM-2 for 24 h. The levels of Pyk2, HO-1, and β-actin (served as an internal control) protein were determined by Western blot. (**D**) Cells were pretreated without or with PF431396 (10 μM), APO (100 μM), DPI (10 μM), or NAC (10 mM) for 1 h, and then incubated with CORM-2 for the indicated time intervals. The levels of phospho-Pyk2 and Pyk2 (served as an internal control) were determined by Western blot. Data are expressed as mean ± SEM of five independent experiments (*n* = 5). ^#^ *p* < 0.05, as compared with the cells exposed to CORM-2 alone. Data analysis and processing are described in the section “Statistical Analysis of Data”.

Previous reports have indicated that PF431396 treatment attenuates proline-rich tyrosine kinase 2 (Pyk2) phosphorylation at Tyr (tyrosine)^402^ in various types of cells [36,37]. To investigate the role of Pyk2 in HO-1 expression, PF431396 (a Pyk2 inhibitor) was used. In this study, we found that pretreatment with PF431396 concentration-dependently attenuated CORM-2-induced both of HO-1 protein (Figure 5A) and mRNA (Figure 5B) expression. To confirm the role of Pyk2 in CORM-2-induced HO-1 expression, Pyk2 siRNA transfection significantly knocked down the Pyk2 protein level and inhibited CORM-2-induced HO-1 expression (Figure 5C). We further determined whether CORM-2 stimulated activation of Pyk2; the phosphorylation of Pyk2 was detected by Western blot. As shown in Figure 5D, CORM-2 time-dependently stimulated Pyk2 phosphorylation, which was attenuated by pretreatment of PF431396 (10 μM), but not by APO, DPI, or NAC, indicating that Pyk2 is an upstream component of Nox/ROS in HO-1 expression. This note was also supported by the results that PF431396 attenuated the CORM-2-triggered Nox activity and ROS generation (Figure 3E,F). These findings indicated that HO-1 expression by CORM-2 is mediated via a Pyk2-dependent Nox/ROS activity in HTSMCs. 

### 2.6. Involvement of ERK1/2 in CORM-2-Induced HO-1 Expression

**Figure 6:**
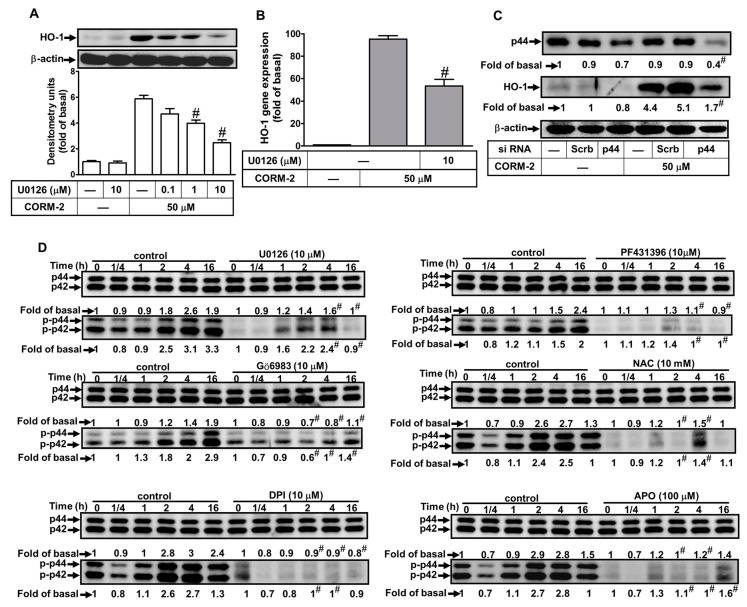
CORM-2-induced HO-1 expression is mediated through extracellular signal-regulated kinase 1/2 (ERK1/2) in HTSMCs. (**A**) Cells were pretreated with U0126 for 1 h, and then incubated with CORM-2 for 24 h. The protein levels of HO-1 and β-actin (served as an internal control) were determined by Western blot. (**B**) Cells were pretreated with U0126 (10 μM) for 1 h, and then incubated with CORM-2 for 6 h. The mRNA expression of HO-1 was determined by real-time PCR. (**C**) Cells were transfected with either scrambled (Scrb) or p44 siRNA, and then incubated with CORM-2 for 24 h. The levels of p44 and HO-1 protein were determined by Western blot. (**D**) Cells were pretreated with or without U0126 (10 μM), Gö6983 (10 μM), PF431396 (10 μM), NAC (10 mM), DPI (10 μM), or APO (100 μM), and then incubated with CORM-2 for the indicated time intervals. The levels of phospho-p42/p44 MAPK and p42/p44 MAPK (served as an internal control) were determined by Western blot. Data are expressed as mean ± SEM of five independent experiments (*n* = 5). ^#^ *p* < 0.05, as compared with the cells exposed to CORM-2 alone. Data analysis and processing are described in the section “Statistical Analysis of Data”.

We have previously demonstrated that MAPKs participate in the HO-1 induction by LTA and CSPE in HTSMCs [20]. Therefore, we further approached the roles of p42/p44 MAPK in CORM-2-induced HO-1 expression in these cells. We found that pretreatment with a MEK1/2 inhibitor (U0126) significantly blocked HO-1 protein and mRNA expressions (Figure 6A,B), which were both CORM-2-induced. To further ensure the role of p42/p44 MAPK in CORM-2-induced HO-1 expression, transfection with p44 siRNA markedly knocked down the p44 protein level and blocked the CORM-2-induced HO-1 expression (Figure 6C). To confirm whether p42/p44 MAPK phosphorylation is necessary for CORM-2-induced HO-1 expression, activation of the kinases was assayed by Western blot using an antibody specific for the phosphorylated form of p42/p44 MAPK. As shown in Figure 6D, CORM-2 stimulated a time-dependent phosphorylation of p42/p44 MAPK, which was inhibited by pretreatment with U0126 (10 μM) during the period of observation. Further, pretreatment with Gö6983, PF431396, NAC, DPI, or APO significantly attenuated CORM-2-stimulated p42/p44 MAPK phosphorylation (Figure 5D), indicating that p42/p44 MAPK was a downstream component of PKCα, which is a Pyk2-mediated Nox/ROS generation pathway. Our findings demonstrated that CORM-2-induced HO-1 expression is mediated through the activation of the PKCα, Pyk2/Nox/ROS/p42/p44 MAPK pathway in HTSMCs. 

### 2.7. Induction of c-Fos and c-Jun/AP-1 is Required for CORM-2-Induced HO-1 Expression

**Figure 7:**
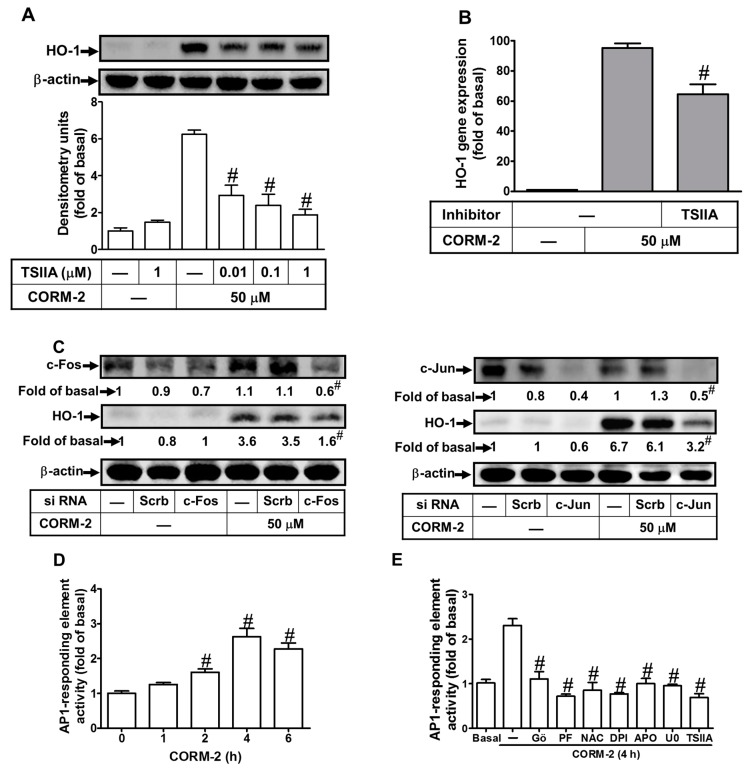
Involvement of c-Fos and c-Jun/activator protein 1 (AP-1) in CORM-2-mediated HO-1 expression. (**A**) Cells were pretreated with tanshinone IIA (TSIIA) for 1 h, and then incubated with CORM-2 for 24 h. The protein levels of HO-1 and β-actin (served as an internal control) were determined by Western blot. (**B**) Cells were pretreated with TSIIA (1 μM) for 1 h, and then incubated with CORM-2 for 6 h. The mRNA expression of HO-1 was determined by real-time PCR. (**C**) Cells were transfected with either scrambled (Scrb), c-Fos, or c-Jun siRNA, and then incubated with CORM-2 for 24 h. The levels of c-Fos, c-Jun, HO-1, and β-actin (served as an internal control) protein were determined by Western blot. (**D**) Cells were transiently cotransfected with pAP1-Luc (activator protein-1 luciferase reporter plasmid) and pGal (plasmid contains the reporter gene β-galactosidase) for 24 h, and then incubated with CORM-2 for the indicated time intervals. (**E**) Cells were pretreated with Gö6983 (10 μM), PF431396 (10 μM), N-acetyl-cysteine (NAC, 10 mM), DPI (10 μM), APO (100 μM), U0126 (10 μM), or TSIIA (1 μM) for 1 h and then incubated with CORM-2 for 4 h. The AP-1 promoter activity in the cell lysates was determined as described in the Methods section. Data are expressed as mean ± SEM of five independent experiments (*n* = 5). ^#^ *p* < 0.05, as compared with the cells exposed to vehicle (**D**) or CORM-2 (**A**–**C**,**E**) alone. Data analysis and processing are described in the section “Statistical Analysis of Data”.

Moreover, AP-1 has been shown to regulate HO-1 expression through binding to respective elements in the promoter region in response to oxidative stress [38,39]. Hence, we determined whether CORM-2-induced HO-1 expression was mediated via AP-1 using its inhibitor tanshinone IIA (TSIIA). As shown in Figure 7A,B, pretreatment with TSIIA attenuated CORM-2-induced HO-1 protein and mRNA expression, suggesting that in HTSMCs, activated AP-1 is an important event for CORM-2-induced HO-1 expression. To further approach the roles of c-Fos and c-Jun in CORM-2-induced HO-1 expression, transfection with either c-Fos or c-Jun siRNA significantly knocked down the c-Fos or c-Jun protein expression, respectively, and attenuated HO-1 induction in CORM-2-treated HTSMCs (Figure 7C). To study whether CORM-2 accelerated AP-1 transcription activity, a reporter plasmid construct containing the AP-1 response element was used to evaluate AP-1 activity in HTSMCs. We found that CORM-2 stimulated a time-dependent AP-1 promoter activity (Figure 7D), which was inhibited by pretreatment with Gö6983, PF431396, NAC, DPI, APO, U0126, or TSIIA (Figure 7E). These results suggested that HO-1 induction is mediated via PKCα, Pyk2/Nox/ROS/p42/p44 MAPK-dependent activation of AP-1(c-Fos/c-Jun) in CORM-2-treated HTSMCs.

### 2.8. CORM-2 Induces HO-1 Expression via Its Promoter Transcriptional Activity

**Figure 8:**
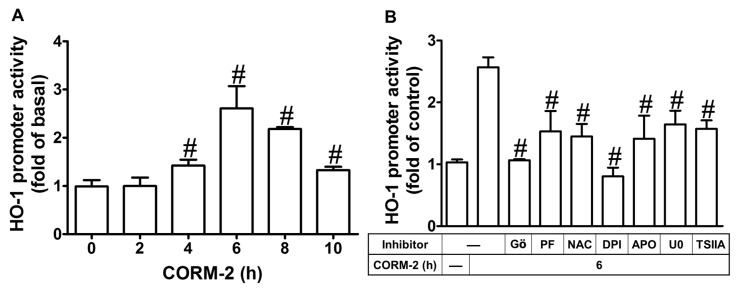
CORM-2 induces AP-1-dependent HO-1 expression via PKCα/Pyk2/Nox/ROS/ERK pathway in HTSMCs. (**A**) Cells were transiently cotransfected with pHO1-Luc and pGal for 24 h, and then incubated with CORM-2 (50 μM) for the indicated time intervals. (**B**) Cells were pretreated with Gö6983 (10 μM), PF431396 (10 μM), NAC (10 mM), DPI (10 μM), APO (100 μM), U0126 (10 μM), or TSIIA (10 μM) for 1 h and then incubated with CORM-2 for 6 h. The HO-1 promoter activity in the cell lysates was determined. Data are expressed as mean ± SEM of five independent experiments (*n* = 5). ^#^ *p* < 0.05, as compared with the cells exposed to vehicle (**A**) or CORM-2 alone. Data analysis and processing are described in the section “Statistical Analysis of Data”.

CORM-2 has been shown to induce HO-1 gene regulation and activate the AP-1 activity. Thus, human HO-1 promoter was constructed in a luciferase reporter plasmid to evaluate the CORM-2 induced HO-1 transcription in HTSMCs. The HO-1 promoter contains several putative recognition elements for a variety of transcriptional factors, including the AP-1 site. As expected, CORM-2 stimulated the HO-1 promoter activity in a time-dependent manner with a maximal response within 6 h (Figure 8A), which was inhibited by pretreatment with Gö6983, PF431396, NAC, DPI, APO, U0126, or TSIIA (Figure 8B). These results confirmed that CORM-2 stimulates HO-1 promoter activity via AP-1 activation, which was mediated through PKCα or Pyk2-regulated Nox/ROS/p42/p44 MAPK-dependent c-Fos-c-Jun/AP-1 pathway in HTSMCs.

## 3. Discussion

**Figure 9:**
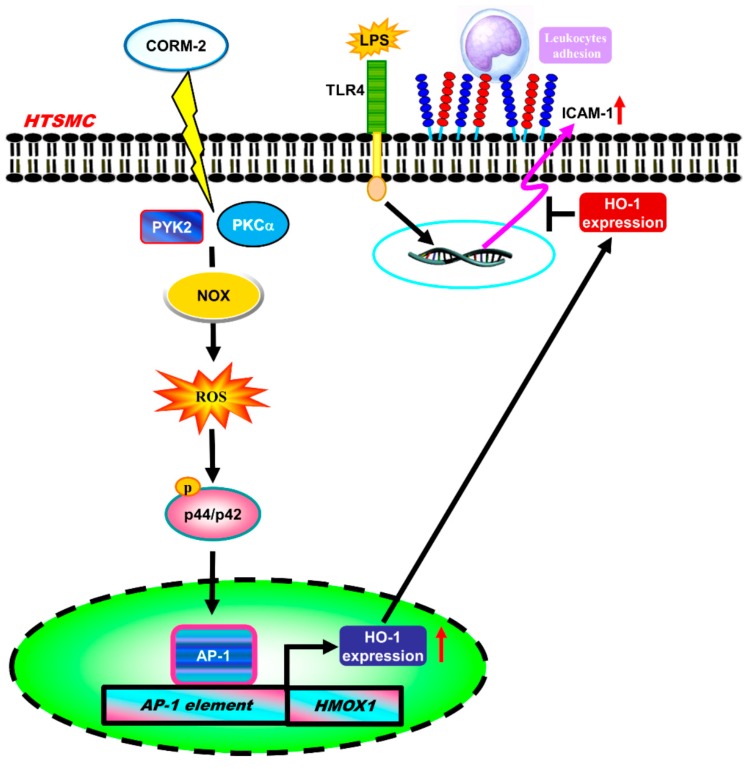
Schematic signaling pathways are involved in CORM-2-induced HO-1 expression in HTSMCs. CORM-2-induced HO-1 expression is mediated via a PKCα or Pyk2/Nox/ROS/ERK1/2 cascade linking to activation of c-Fos and c-Jun/AP-1. The up-regulation of HO-1 could protect against the LPS-induced airway inflammation.

CORMs exert anti-inflammatory effects through the up-regulation of anti-oxidant enzymes such as HO-1 [26]. However, the roles of Nox/ROS involved in CORM-2-induced HO-1 expression were still unknown. Here, we observed that pretreatment with CORM-2 inhibited the LPS-induced airway inflammation via HO-1 induction in mice. Further, our results demonstrated that the levels of HO-1 protein, mRNA, and promoter activity were increased in HTSMCs challenged with CORM-2. CORM-2-induced HO-1 expression was mediated through PKCα and Pyk2/Nox/ROS/ERK1/2 linking to the AP-1 pathway in HTSMCs (Figure 9). These results suggested that HO-1 expression induced by CORM-2 is mediated via a PKCα and Pyk2/Nox/ROS/p42/p44 MAPK-dependent AP-1 pathway in HTSMCs.

ROS exert as a messenger in the normal physiological functions and the inflammatory responses dependent on their cellular concentrations [40]. The up-regulation of HO-1 due to Nox activity and ROS formation is induced by LPS and cytokines [8,19]. Others and our previous studies indicated that the CORMs mediate Nox-dependent ROS generation in astrocytes [21,22]. Therefore, Nox-dependent ROS generation is involved in HO-1 expression by CORM-2 in HTSMCs. We further clarified the role of ROS in HO-1 expression; a thiol-containing compound (NAC) was used to scavenge ROS. NAC has been shown to reduce the injurious effects of hydrogen peroxide in human alveolar and bronchial epithelial cells [41]. We also found that CORM-2-induced HO-1 expression was inhibited by NAC, and strongly supported the role of ROS in the CORM-2-induced HO-1 expression. 

Moreover, two Nox-related inhibitors, DPI (a Nox inhibitor) and APO (a p47*^phox^* inhibitor), have been shown to prevent p47*^phox^* (a Nox subunit) translocation to the membrane and inhibit Nox activation [42]. Our results showed that pretreatment with either DPI or APO attenuated CORM-2-induced ROS generation and HO-1 expression. These data are consistent with previous reports showing that Nox-derived ROS generation is involved in HO-1 induction by LTA or CSPE in HTSMCs [5,20]. Indeed, low levels of ROS could regulate proliferation, gene expression, immunity, and wound healing [43]. Conversely, higher levels of ROS can exert antibacterial effect, and cause cell damage and death [23,44]. In addition, ROS generation could initiate HO-1 expression through the degradation of Keap1 and translocation of Nrf2 into the nucleus. In our previous study, CORM-2 has been shown to activate Nox and produce ROS in brain astrocytes [22]. Previous reports also indicate that CO release in mammal cells acts as a secondary messenger to mediate metabolism and gene expression, including HO-1 [45,46]. The members of Nox family such as Nox-(1,2,4) and p47*^phox^* were shown to be involved in CORM-2-induced HO-1 expression, which were confirmed by using their own siRNAs. In this study, we also demonstrated that PKCα and Pyk2 are the upstream components of ROS generation by their inhibitors. Our results showed that PKCα/Pyk2-mediated Nox/ROS signal contributes to CORM-2-induced HO-1 expression in HTSMCs. However, how CORM-2 activated PKCα/Pyk2 and led to Nox/ROS generation is an important issue preserved for further investigation. 

Abnormal MAPK activations are implicated in a variety of inflammatory responses and tissue injury, and the induction of several inflammatory mediators in different cell types [25,47]. Here, we demonstrated that ERK1/2 was required for the CORM-2-induced HO-1 expression, which was attenuated by a selective MEK1/2 inhibitor U0126 or transfection with p44 siRNA. These kinases involved in CORM-2-stimulated pathways were further confirmed by CORM-2-mediated ERK1/2 phosphorylation. These results are consistent with the HO-1 expression mediated by ERK1/2 to activate the antioxidant response element (ARE) region, which is the Nrf2 binding site in HepG2 (liver hepatocellular carcinoma) Cells [48] and heme-mediated neuronal injury [49]. Moreover, pretreatment with the inhibitor of PKCα, Pyk2, Nox, or ROS scavenger significantly attenuated ERK1/2 phosphorylation, suggesting that PKCα/Pyk2-dependent Nox/ROS are required for ERK1/2 phosphorylation. These results are consistent with reports that ROS-dependent MAPK pathways are involved in the regulation of cellular functions [38,50,51]. Indeed, our previous study found that the inhibition of JNK1/2 or p38 MAPK attenuates CORM-2-induced HO-1 expression via a c-Src/EGFR/PI3K/Akt pathway [27]. In contrast, CORM-2-stimulated JNK1/2 and p38 MAPK phosphorylation was not mediated via the PKCα/Pyk2-mediated Nox/ROS signal, although these two kinases also regulate CORM-2-induced HO-1 expression in HTSMCs. 

The activated transcription factors interact with response elements on the HO-1 promoter to regulate gene transcription [44]. Here, we focused on the role of transcription factor AP-1, which is modulated during oxidative stress associated with inflammatory diseases [52]. The involvement of AP-1 in these responses was further supported by the results that CORM-2 induced c-Fos and c-Jun, AP-1 subunits, and activation via PKCα/Pyk2-mediated Nox/ROS linking to the ERK1/2 pathway. Moreover, the roles of c-Fos-c-Jun/AP-1 in CORM-2-induced HO-1 expression were confirmed by transfection with c-Fos or c-Jun siRNA to attenuate CORM-2-induced HO-1 expression. Several reports have shown that many regulatory elements of transcription factors, including AP-1, were analyzed on the 5′ region of the HO-1 promoter in several animal species [28,53]. Thus, we also demonstrated that CORM-2-stimulated HO-1 promoter activity was reduced by pretreatment with Gö6976, PF431396, NAC, DPI, APO, U0126, or TSIIA, indicating that CORM-2 induces HO-1 promoter activity via a PKCα/Pyk2-mediated Nox/ROS/ERK1/2/AP-1 pathway. These results are consistent with the reports that alpha-lipoic acid induced HO-1 expression in vascular smooth muscle cells [54], and BK induced HO-1 expression in brain astrocytes [38]. 

Previous reports indicated that the CORMs up-regulate the HO-1 activity and attenuate the LPS-induced inflammatory responses in macrophages [9] and animal study [8,31]. Moreover, the overexpression of HO-1 in ovalbumin (OVA)-sensitized guinea pigs effectively decreases inflammatory reaction, mucus secretion, and responsiveness to histamine in airways [55], suggesting that HO-1 exhibits protecting ability in the host during airway inflammation. In this study, the induction of HO-1 by CORM-2 protected against LPS-induced ICAM-1 expression and leukocytes infiltration in both in vitro and in vivo studies. Importantly, previous reports also indicated that CORM-2-derived CO release can attenuate the cell sequestration, NF-κB activity, and ICAM-1 expression of leukocyte after lung injury [36,56] and regulate the expressions of adhesion molecules on human umbilical vein endothelia cells to affect leukocyte attachment [54]. Our previous report also indicated that the induction of HO-1 by CoPPIX inhibits the TNF-α-induced ICAM-1 and VCAM-1 expression which is revered by zinc protoporphyrin IX (ZnPPIX, an inhibitor of HO-1 activity) in HTSMCs [14].

## 4. Materials and Methods

### 4.1. Reagents and Chemicals

DMEM/F-12 (Dbecco’s Modified Eagle Medium/Nutrient Mixture F-12) medium, fetal bovine serum (FBS), TRIzol reagent, and PLUS-Lipofectamine were from Invitrogen (Carlsbad, CA, USA). Human siRNAs for PKCα (L-003523-00-0020) was from Dharmacon (Lafayette, CO, USA) and Pyk2 (SASI_Hs01_00032249), ERK1 (SASI_Hs01_00190617), HO-1 (SASI_Hs01_00035065), Nox-1 (SASI_Hs01_00342845), Nox-2 (SASI_Hs01_00086110), Nox-4 (SASI_Hs02_00349918), p47*^phox^* (SASI_Hs02_00302212), and c-Fos (SASI_Hs01_00184572) were from Sigma (St. Louis, MO, USA). Hybond C membrane, enhanced chemiluminescence (ECL), and Western blotting detection system were from GE Healthcare Biosciences (Buckinghamshire, UK). PhosphoPlus PKCα (#9375), Pyk2 (#3291), and ERK1/2 (#9101) antibodies were from Cell Signaling (Danvers, MA, USA). The HO-1 (ADI-SPA-895) antibody was from Enzo (Farmingdale, NY, USA). PKCα (sc-208), Pyk2 (sc-9019), ERK1 (sc-94), c-Fos (sc-7202), and β-actin (sc-47778) antibodies were from Santa Cruz (Santa Cruz, CA, USA). Ro31-8220, Gö6983, Gö6976, PF431396, diphenyleneiodonium chloride (DPI), apocynin (APO), U0126, and tanshinone IIA (TSIIA) were from Biomol (Plymouth Meeting, PA, USA). The bicinchoninic acid (BCA) protein assay kit was from Pierce (Rockford, IL, USA). SDS-PAGE (sodium dodecyl sulfate polyacrylamide gel electrophoresis) reagents were from MDBio Inc (Taipei, Taiwan). Tricarbonyldichlororuthenium (II) dimer (CORM-2), ruthenium (III) chloride, RuCl_3_ [inactive form of CORM-2, (iCORM-2)], N-acetyl-cysteine (NAC), lipopolysaccharide (LPS), enzymes, and other chemicals were from Sigma (St. Louis, MO, USA).

### 4.2. Animal Care and Experimental Procedures

Male ICR mice aged 6–8 weeks were purchased from the National Laboratory Animal Centre (Taipei, Taiwan) and handled according to the guidelines of Animal Care Committee of Chang Gung University (Approval Document No. Chang Gung University 16-046, 4 October 2016) and National Institute of Health (NIH) Guides for the Care and Use of Laboratory Animals. All the studies involving animals are reported in accordance with the ARRIVE guidelines [57,58]. Mice were assigned randomly into three groups: sham [0.1 mL of dimethyl sulfoxide (DMSO)-phosphate-buffered saline (PBS) (1:100) with 0.1% (*w*/*v*) bovine serum albumin (BSA) treated mice], LPS (LPS-treated mice), and CORM-2 + LPS; 5 mice in each group/cage and kept in standard individually ventilated cages in an animal facility under standardized conditions (12 h light/dark cycle, 21–24 °C, humidity of 50–60%) with food and water ad libitum. Mice were intraperitoneally (i.p.) injected with CORM-2 (8 mg/kg of body weight) for 24 h, and then anesthetized by i.p. injection of pentothal (50 mg/kg) placed individually on a board in a near-vertical position and the tongues withdrawn with a lined forceps. LPS (3 mg/kg) was placed posterior in the throat and aspirated into lungs for 16 h of development of a lung inflammation model [59]. At the end of the experimental period, mice were killed by a high dose of pentothal (100 mg/kg i.p.) for the collection of lung tissues extracted for protein (right superior lobe + post caval lobe) and mRNA (right middle lobe + right inferior lobe) expression of ICAM-1, HO-1, or β-actin analyses. BAL fluid was performed through a tracheal cannula using 1-mL aliquots of ice-cold PBS solution. BAL fluid was centrifuged at 500× *g* at 4 °C, and cell pellets were washed and re-suspended in PBS. Leukocyte count was determined by a hemocytometer, as previously described [7]. Data collection and evaluation of all the in vivo and in vitro experiments were performed blindly of the group identity.

### 4.3. Cell Culture and Treatment 

HTSMCs were purchased from ScienCell Research Laboratories (San Diego, CA, USA). The cultured conditions and treatments were conducted as previously described [60]. Cells were plated onto 12-well culture plates and made quiescent at confluence by incubation in serum-free DMEM/F-12 for 24 h. Growth-arrested cells were incubated with or without CORM-2 at 37 °C for the indicated time intervals. Our previous report indicates that CORM-2 induces HO-1 expression in time-dependent and concentration-dependent manners. The HO-1 expression is up-regulated to a maximal response within 16–24 h treatment with 50 μM of CORM-2 [27]. Therefore, the concentration of CORM-2 at 50 μM was used throughout this study. When the inhibitors were used, cells were pretreated with the inhibitor for 1 h before exposure to CORM-2. Experiments were performed using cells from passages four to seven. 

### 4.4. Preparation of Cell Extracts and Western Blot Analysis 

After treatment, the cells were washed with ice-cold PBS, scraped, and collected by centrifugation at 16,000× *g* for 10 min at 4 °C to yield the whole cell extract, as previously described [7]. The supernatants were harvested and mixed with SDS-PAGE loading buffer (final concentration: 100 mM Tris-HCl pH 6.8, 1% SDS, 2.5% glycerol, 100 mM β-mercaptoethanol, 0.01% bromophenol blue). Samples were denatured, separated with 10% SDS-PAGE, and transferred to the nitrocellulose membrane. Membranes were probed overnight with an anti-phospho-PKCα, phospho-Pyk2, phospho-ERK1/2, HO-1, PKCα, Pyk2, ERK1, c-Fos, or β-actin antibody. Membranes were washed with Tween-Tris buffered solution (TTBS) four times for 5 min each, incubated with anti-rabbit or anti-mouse horseradish peroxidase antibody (1:2000) for 1 h. The immunoreactive bands were detected by ECL (enhanced chemiluminescence) reagents and captured by a UVP BioSpectrum 500 Imaging System (Upland, CA, USA). The image densitometry analysis was conducted using UN-SCAN-IT gel software (Orem, UT, USA).

### 4.5. Total RNA Extraction and Real Time-Quantitative PCR Analysis 

Total RNA was isolated from HTSMCs treated with CORM-2 for the indicated time intervals in 10-cm culture dishes with TRIzol according to the protocol of the manufacturer. The mRNA was reverse-transcribed into cDNA and analyzed by real time-quantitative (q)PCR. Real time-qPCR was performed with the TaqMan gene expression assay system, using primers and probe mixes for HO-1, c-Fos, and endogenous GAPDH control genes. PCRs were performed using a 7500 Real Time-PCR System (Applied Biosystems, Foster City, CA, USA). Relative gene expression was determined by the ΔΔCt method, where Ct meant threshold cycle. All the experiments were performed in triplicate.

### 4.6. Plasmid Construction, Transfection, and Luciferase Reporter Gene Assays 

For construction of the HO-1 luciferase (Luc) plasmid, human HO-1 promoter, a region spanning –3106 to +186 bp provided by Dr. Y. C. Liang (Graduate Institute of Biomedical Technology, Taipei Medical University, Taipei, Taiwan) was inserted into a pGL3-basic vector (Promega, Madison, WI, USA). Plasmid pAP1-Luc, the fragment of the AP-1-responding element was inserted into the pGL3 (plasmid contains the reporter gene β-galactosidase) promoter. The plasmid DNA was extracted by using QIAGEN plasmid DNA preparation kits and transfected into HTSMCs with Lipofectamine reagent according to the standard protocol of the manufacturer. The plasmid pCMV-β-gal was cotransfected to be the internal control. The HO-1 promoter-driven-Luc activity was analyzed by a luciferase assay system (Promega, Madison, WI, USA). Firefly luciferase activities were standardized with β-galactosidase activity.

### 4.7. Transient Transfection with siRNAs 

HTSMCs (3 × 10^5^ cells) were plated in 12-well culture plates for 24 h to about 80% confluence. Cells were washed once with PBS, and 0.4 mL of serum-free DMEM/F-12 medium was added to each well. The transient transfection of siRNAs (scrambled, PKCα, Pyk2, ERK1, and c-Fos, 100 nM) was performed by using Lipofectamine^TM^ RNAiMAX reagent (from Sigma, St. Louis, MO, USA) according to the manufacturer’s instructions. 

### 4.8. Measurement of Intracellular ROS Generation 

The peroxide-sensitive fluorescent probe CMH_2_DCF-DA and DHE were used to assess the intracellular ROS generation [61] with minor modifications. Briefly, HTSMCs were incubated with 10 μM of CMH_2_DCF-DA (in warm PBS) for 45 min at 37 °C. The medium was removed and replaced with fresh DMEM/F-12 media for CORM-2 treatments. CMH_2_ DCF-DA interacted with cells, and then generated a non-fluorescent product: H_2_DCF. CORM-2 induced the generation of a ROS oxidized product: DCF. Relative fluorescence intensity was recorded (0.5 to 24 h) by a fluorescent plate reader (Thermo, Appliskan; Waltham, MA, USA) at an excitation wavelength of 485 nm, and emission was measured at a wavelength of 530 nm. For DHE staining, cells were treated with CORM-2 for the indicated time intervals and then incubated with 10 μM of DHE (in DMEM/F12 medium) for 10 min. For immunofluorescence staining, the stained cells were washed three times with cold PBS, and then the fluorescence for DCF and DHE staining was detected at 495/529 and 518/605 nm, respectively, using a fluorescence microscope (Zeiss, Axiovert 200M; Oberkochen, Baden-Württemberg, Germany). 

### 4.9. Determination of NADPH Oxidase Activity by Chemiluminescence Assay 

The Nox activity in intact cells was assayed by lucigenin chemiluminescence [38]. After incubation, the cells were gently scraped and centrifuged at 400× *g* for 10 min at 4 °C. The cell pellet was re-suspended in a known volume (35 μL/well) of ice-cold RPMI 1640 medium, and the cell suspension was kept on ice. To a final 200 μL of pre-warmed (37 °C) PBS containing either NADPH (1 μM) or lucigenin (20 μM), 5 μL of cell suspension (2 × 10^4^ cells) was added to initiate the reaction followed by the immediate measurement of chemiluminescence using an Appliskan luminometer (Thermo^®^; Waltham, MA, USA) in an out-of-coincidence mode. Neither NADPH nor NADH enhanced the background chemiluminescence of lucigenin alone (30–40 counts/min). Chemiluminescence was continuously measured for 12 min, and the activity of Nox was expressed as counts per million cells. The calculated numbers of Nox activity were calibrated with protein concentration. The equal amount of warmed PBS medium (containing NADPH and lucigenin) was used as the blank, and the untreated cells were the basal group.

### 4.10. Statistical Analysis of Data 

All the data were expressed as the mean ± SEM in at least five individual experiments (*n* = 5). Statistical analysis was performed by using GraphPad Prizm Program 6.0 software (GraphPad, San Diego, CA). We used one-way ANOVA followed by Dunnett’s post hoc test when comparing more than two groups of data and a one-way ANOVA, non-parametric Kruskal–Wallis test, followed by Dunnett’s post hoc test when comparing multiple independent groups. *p* values of 0.05 were considered to be statistically significant. Post tests were run only if F achieved *p* < 0.05 and there was no significant variance in homogeneity. Error bars were omitted when they fell within the dimensions of the symbols. 

## 5. Conclusions

These results suggested that CORM-2-induced HO-1 expression is mediated through PKCα/Pyk2 and Nox/ROS-dependent activation of ERK1/2, linking to the up-regulation of AP-1 (c-Fos and c-Jun), which promotes HO-1 expression and enzymatic activity in HTSMCs. Based on the observations from literatures and our findings, we depict a model for the molecular mechanisms underlying CORM-2-induced HO-1 expression and activity in HTSMCs. The results obtained with cellular and animal experiments indicated that better understanding the mechanisms underlying CORM-2-induced HO-1 expression promotes the development of therapeutic strategies for airway inflammatory disorders.

## Abbreviations

AP-1	activator protein 1
APO	apocynin
ARE	antioxidant response element
BAL	bronchoalveolar lavage
BCA	bicinchoninic acid
BSA	Bovine serum albumin
CO	carbon monoxide
CORM-2	carbon monoxide releasing molecule-2
CoPPIX	cobalt protoporphyrin
CSPE	cigarette smoke particle extract
DCF	dichlorofluorescein
DCF-DA	2',7'-dichlorofluorescin diacetate
DHE	dihydroethidium
DMEM/F-12	Dubecco’s Modified Eagle Medium/Nutrient Mixture F-12
DMSO	dimethyl sulfoxide
DPI	diphenyleneiodonium
ECL	enhanced chemiluminescence
EGFR	epidermal growth factor receptor
ERK1/2	extracellular signal-regulated kinase 1/2
FBS	fetal bovine serum
MCH_2_DCF-DA	chloromethyl 2’,7’-dichloro fluorescein diacetate
H & E	hematoxylin & eosin
HO-1	heme oxygenase-1
HTSMCs	human tracheal smooth muscle cells
ICAM-1	intercellular adhesion molecule
ICR	Institute of Cancer Research
i.p.	intraperitoneally
LPS	lipopolysaccharide
LTA	lipotechoic acid
MAPKs	mitogen-activated protein kinases
mRNA	messenger ribonucleic acid
NAC	N-acetyl-cysteine
NADPH	nicotinamaide adenine dinucleotide phosphate
NF-κB	nuclear factor-κB
Nox	NADPH oxidase
Nrf2	nuclear factor erythroid 2-related factor 2
PBS	phosphate-buffered saline
PI3K	phosphoinositide 3-kinase
PKC	protein kinase C
Pyk2	proline-rich tyrosine kinase 2
ROS	reactive oxygen species
SDS-PAGE	sodium dodecyl sulfate polyacrylamide gel electrophoresis
siRNA	small interfering ribonucleic acid
TNF	tumor necrosis factor
TSIIA	tanshinone IIA
TTBS	Tween-Tris buffered solution
VCAM-1	vascular cell adhesion molecule
XTT	2,3-bis-(2-methoxy-4-nitro-5-sulfophenyl)-2H-tetrazolium-5-carboxanilide
ZnPP IX	zinc protoporphyrin IX
